# Identification of conformational epitopes for human IgG on Chemotaxis inhibitory protein of *Staphylococcus aureus*

**DOI:** 10.1186/1471-2172-10-13

**Published:** 2009-03-11

**Authors:** Erika Gustafsson, Pieter-Jan Haas, Björn Walse, Marcel Hijnen, Christina Furebring, Mats Ohlin, Jos AG van Strijp, Kok PM van Kessel

**Affiliations:** 1 Alligator Bioscience AB, Scheelev. 19A, S-223 70 Lund, Sweden; 2 Alligator Bioscience AB, S-223 70 Lund, Sweden; 3 Department of Immunotechnology, Lund University, BMC D13, S-221 84 Lund, Sweden; 4 Medical Microbiology, University Medical Center Utrecht, 3584 CX Utrecht, the Netherlands; 5 SARomics AB, S-220 07 Lund, Sweden; 6 Macfarlane Burnet Institute for Medical Research and Public Health, Melbourne, Victoria 3004, Australia

## Abstract

**Background:**

The Chemotaxis inhibitory protein of *Staphylococcus aureus *(CHIPS) blocks the Complement fragment C5a receptor (C5aR) and formylated peptide receptor (FPR) and is thereby a potent inhibitor of neutrophil chemotaxis and activation of inflammatory responses. The majority of the healthy human population has antibodies against CHIPS that have been shown to interfere with its function *in vitro*. The aim of this study was to define potential epitopes for human antibodies on the CHIPS surface. We also initiate the process to identify a mutated CHIPS molecule that is not efficiently recognized by preformed anti-CHIPS antibodies and retains anti-inflammatory activity.

**Results:**

In this paper, we panned peptide displaying phage libraries against a pool of CHIPS specific affinity-purified polyclonal human IgG. The selected peptides could be divided into two groups of sequences. The first group was the most dominant with 36 of the 48 sequenced clones represented. Binding to human affinity-purified IgG was verified by ELISA for a selection of peptide sequences in phage format. For further analysis, one peptide was chemically synthesized and antibodies affinity-purified on this peptide were found to bind the CHIPS molecule as studied by ELISA and Surface Plasmon Resonance. Furthermore, seven potential conformational epitopes responsible for antibody recognition were identified by mapping phage selected peptide sequences on the CHIPS surface as defined in the NMR structure of the recombinant CHIPS_31–121 _protein. Mapped epitopes were verified by *in vitro *mutational analysis of the CHIPS molecule. Single mutations introduced in the proposed antibody epitopes were shown to decrease antibody binding to CHIPS. The biological function in terms of C5aR signaling was studied by flow cytometry. A few mutations were shown to affect this biological function as well as the antibody binding.

**Conclusion:**

Conformational epitopes recognized by human antibodies have been mapped on the CHIPS surface and amino acid residues involved in both antibody and C5aR interaction could be defined. This information has implications for the development of an effective anti-inflammatory agent based on a functional CHIPS molecule with low interaction with human IgG.

## Background

Chemotaxis inhibitory protein of *Staphylococcus aureus *(CHIPS) is a potent inhibitor of neutrophil chemotaxis and activation by specifically binding and blocking the G-protein coupled Complement fragment C5a receptor (C5aR) and formylated peptide receptor (FPR) [1,2]. C5a is a complement polypeptide with many functions. It exerts pro-inflammatory effects through the C5aR and is involved in host defense against microorganisms. The C5a/C5aR interaction mediates immunomodulatory and inflammatory activities such as chemotaxis, degranulation, vascular permeabilisation and cytokine regulation [3-6]. C5a plays a role in a wide variety of inflammatory disorders like rheumatoid arthritis, inflammatory bowel disease, immune complex disease, ischemia-reperfusion injury and sepsis [7-13]. Since C5a is generated early in the inflammatory cascade it is a promising target for anti-inflammatory therapy. Several studies have demonstrated the beneficial effects of targeting the C5aR in inflammatory diseases [14-19].

The potent ability of CHIPS to inhibit activation of the C5aR is an important asset which might be useful for development of a potential new anti-inflammatory drug. However, since the CHIPS gene is present in over 60% of *Staphylococcus aureus *strains and *S. aureus *is a common bacterium, a majority of the population encounters CHIPS early in life. Human serum contains anti-CHIPS antibodies that have been shown to interfere with CHIPS function *in vitro *[20]. These antibodies may therefore neutralize the *in vivo *effect of CHIPS or give rise to antibody mediated immune reactions. As such, the potential of CHIPS to function as an anti-inflammatory molecule is hampered. An improved CHIPS molecule will be characterized by decreased reactivity with pre-existing antibodies, but preserved C5aR blocking activity. Mapping the epitopes of human IgG on the CHIPS protein is an important step in the understanding of how antibodies interfere with CHIPS.

Antibody screening of phage-displayed peptide libraries is a useful approach to identify antibody epitopes [21,22]. Previous studies showed the potential of using random peptide phage libraries in identifying linear epitopes and conformational epitopes for monoclonal and polyclonal antibodies. For example, Luzzago *et al. *identified discontinuous epitopes in human H-subunit ferritin by the use of phage display and verified the potential epitopes by design of variants with point mutations [23]. Rowley *et al. *studied autoantibodies in primary biliary cirrhosis and could predict the major conformational antibody epitope using phage display and the known NMR structure of the target protein [24]. These studies show that peptides expressed by phage display are capable of adopting a conformation that mimics the epitopes.

In this paper we focus on mapping epitopes on CHIPS by panning of phages displaying 7-mer peptides against polyclonal human IgG affinity-purified on N- and C-terminally truncated CHIPS. Peptides selected by phage display could be grouped into two distinct groups based on their sequence. These were then verified in ELISA and by the use of a synthetic peptide. By mapping the selected peptide sequences on the CHIPS surface using the known NMR structure of CHIPS_31–121 _[25], potential conformational epitopes for human IgG could be identified. The epitopes were subsequently verified by mutation of key amino acids in the CHIPS molecule and the activity of the mutants was studied by C5aR inhibition. The mutational analysis of the epitopes revealed amino acid residues that are important for the interaction of CHIPS with human antibodies. In addition, amino acids involved in the interaction with the C5aR were identified.

## Results

### Truncated CHIPS variants inhibit C5a induced cell activation

**Figure 1:**
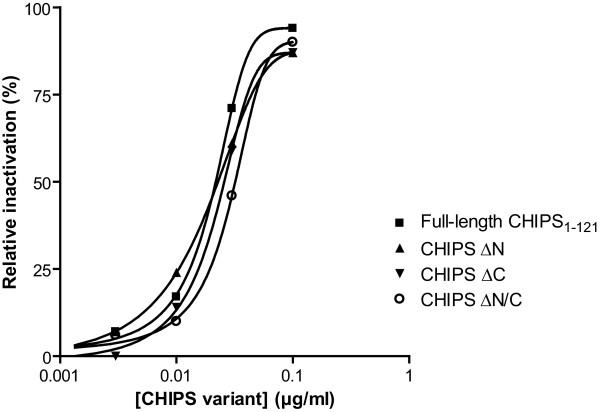
**Truncated CHIPS variants inhibit C5a induced cell activation**. Fluo-3AM labeled U937/C5aR transfectants were preincubated with full-length CHIPS_1–121_, CHIPS with N-terminal truncation (CHIPS ΔN), CHIPS with C-terminal truncation (CHIPS ΔC) and CHIPS with truncations in both the N- and C-terminal ends (CHIPS ΔN/C) and stimulated with 1 nM C5a. Results are expressed as percentage inhibition of buffer treated cells and are from a representative experiment.

In order to isolate a small active CHIPS variant able to form a functional molecule with decreased number of antibody epitopes, we generated truncated variants of CHIPS. Previously, we described CHIPS with N-terminal truncation (ΔN) [25] and CHIPS with both N- and C-terminal truncations (ΔN/C) [26] that showed a complete preservation of C5aR blocking activity. Figure 1 summarizes the activity of different CHIPS variants (CHIPS ΔN, CHIPS ΔC and CHIPS ΔN/C in comparison to full-length CHIPS_1–121_. All truncated CHIPS variants were able to inhibit the C5a induced activation of U937/C5aR cells to the same extent as full-length wild-type (wt) CHIPS_1–121_. Hence, the smallest CHIPS variant tested, a molecule with preserved biological activity with truncations in both the N- and C-terminal end (resulting in a protein of 83 amino acids) was used for further studies. This CHIPS variant has most likely lost the ability to block activation of the FPR, since the N-terminal amino acids of CHIPS are required for that action [27].

### Affinity-purified anti-CHIPS antibodies recognize non-linear epitopes

**Figure 2:**
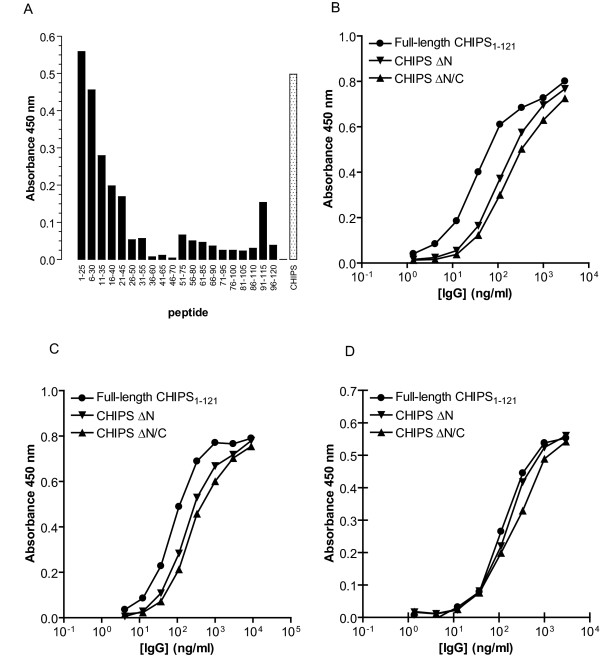
**Affinity-purified anti-CHIPS IgG recognize non-linear epitopes**. Affinity-purified anti-CHIPS_1–121 _IgG was tested for the ability to bind CHIPS derived 25-mer peptides or full-length CHIPS in ELISA (A). Full-length CHIPS_1–121 _or CHIPS with N-terminal truncation (CHIPS ΔN), or CHIPS with truncations in both the N- and C-terminal ends (CHIPS ΔN/C) were compared for reactivity with different human affinity-purified anti-full-length CHIPS_1–121 _IgG (B), anti-CHIPS ΔN/C IgG (C), and the CHIPS specific mouse monoclonal antibody 2G8 as control (D).

In order to map antibody epitopes on CHIPS, affinity purification of human anti-CHIPS IgG was initially performed on the wt CHIPS_1–121 _protein. Pooled human IgG (IV-IgG) was affinity-purified using a column packed with immobilized CHIPS_1–121 _resin. We tested the binding of affinity-purified anti-CHIPS_1–121 _antibodies to a set of CHIPS derived 25-mer peptides spanning the complete CHIPS_1–121 _sequence. As shown in Figure 2A, linear epitopes do appear to be confined to the N-terminus of CHIPS. To confirm the presence of conformational epitopes, we tested the reactivity of two different affinity-purified antibody preparations (anti-full-length CHIPS_1–121 _and anti-CHIPS ΔN/C) to wt CHIPS_1–121_, CHIPS ΔN and CHIPS ΔN/C. Figure 2B–D shows that both preparations of affinity isolated antibodies react with all three CHIPS variants. There is no significant difference in reactivity towards the different CHIPS variants between the preparations. Reactivity of the different CHIPS variants with the control antibody 2G8 (a monoclonal mouse anti-CHIPS antibody [27]) was also comparable, indicating that all CHIPS variants coated equally well. These data also imply that epitopes outside the N-terminus may be of importance for antibody binding and that some of these epitopes are conformational.

### Panning experiments identified potential antibody recognition sequences

**Figure 3:**
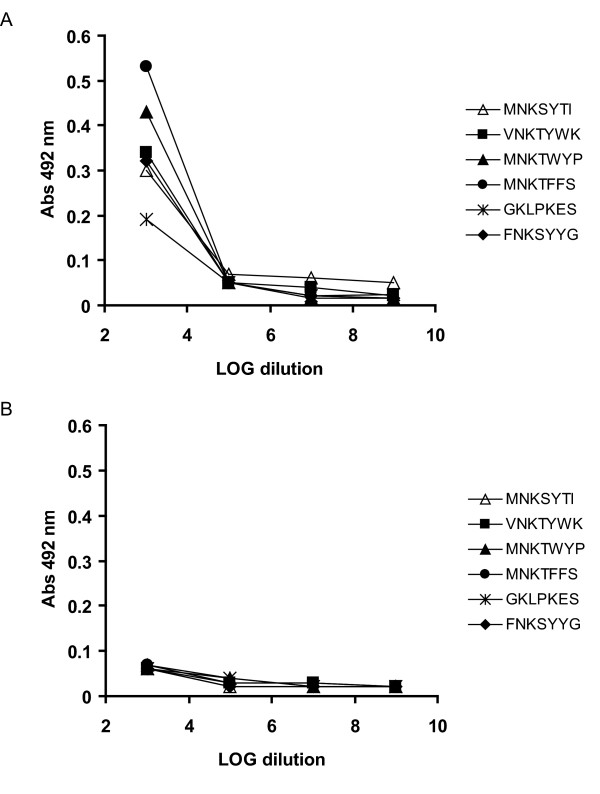
**Selected phages bind human anti-CHIPS ΔN/C IgG in ELISA**. Six different recombinant phages expressing representative peptide sequences were characterized in ELISA for binding to human anti-CHIPS ΔN/C IgG. These phages were found to bind affinity-purified human anti-CHIPS ΔN/C IgG (A) but not BSA (B).

To obtain peptides mimicking epitopes other than the linear epitopes found in the N-terminus of CHIPS, two random peptide phage libraries were screened for binding to anti-CHIPS ΔN/C IgG. The starting material was a mixture of two different libraries (PhD-7 displaying linear peptides and PhD-C7C displaying cyclic peptides). After four rounds of panning, 48 recombinant phage clones were randomly selected and characterized by DNA sequencing. The sequence 'MNKTWYP' occurred 12 times among the sequenced clones and was thereby the most abundant followed by 'MNKTFWF' which was selected five times. A majority of the selected peptides (42 of 48) could be divided into two different groups based on their amino acid sequence as shown in Table 1. Amino acids that occurred most frequently among the aligned sequences for each group were classified as consensus residues. 36 sequences belonged to the first group and their consensus sequence was defined as 'MNKT/Sxxx'. Six sequences with the consensus sequence 'GKLPxxx' belonged to the second group. Although we started out with a mixture of two different libraries (PhD-7 and PhD-C7C) with linear and circular peptides displayed, respectively, the selected sequences were all originating from the PhD-7 library with linear sequences. Six different recombinant phages expressing representative peptide sequences were characterized in ELISA for binding to human anti-CHIPS ΔN/C IgG. These phages bound specifically to the antibodies and not to BSA (Figure 3). We therefore conclude that the binding of the selected phages to affinity-purified human anti-CHIPS ΔN/C IgG is specific for the expressed peptide.

**Table 1:** Selected phage peptide sequences were divided into two groups based on their amino acid sequences*.

***Group 1***	**M**	**N**	**K**	**T/S**	**x**	**x**	**x**	***Frequency***
	M	N	K	T	W	Y	P	(12)
	M	N	K	T	F	W	F	(5)
	M	N	K	T	F	F	S	(2)
	M	N	K	S	Y	H	L	(2)
	M	N	K	T	F	S	A	(1)
	M	N	K	T	F	V	D	(1)
	M	N	K	T	F	V	P	(1)
	M	N	K	V	Y	L	P	(1)
	M	N	K	S	Y	T	I	(1)
	M	N	K	Y	H	N	P	(1)
	F	N	K	S	Y	Y	G	(3)
	Y	N	K	S	F	F	M	(2)
	Y	N	K	S	F	F	P	(1)
	F	N	K	S	W	F	P	(1)
	L	N	K	T	F	Y	Y	(1)
	V	N	K	T	Y	W	K	(1)

***Group 2***	**G**	**K**	**L**	**P**	**x**	**x**	**x**	

	G	K	L	P	I	A	M	(1)
	G	K	L	P	W	P	K	(1)
	G	K	L	P	I	P	Y	(1)
	G	K	L	P	P	P	I	(1)
	G	K	L	P	K	M	T	(1)
	G	K	L	P	K	E	S	(1)

* A consensus sequence for each group was determined and is shown in bold. The frequency of each peptide is shown in parenthesis.

### Synthetic peptides can be used to purify CHIPS specific antibodies

**Figure 4:**
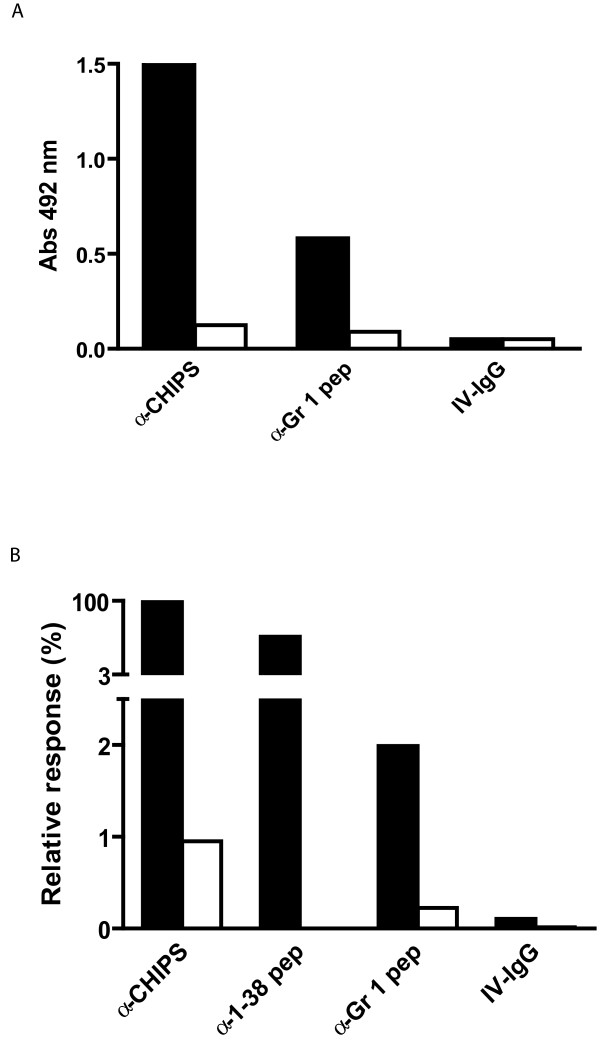
**Synthetic peptides can be used to purify CHIPS specific antibodies**. Binding of affinity-purified anti-CHIPS ΔN/C IgG (a-CHIPS), anti-group 1 peptide (a-Gr 1 pep) and IV-IgG to immobilized CHIPS_1–121 _were measured in ELISA (A) and SPR (B). Black bars represent binding of the different affinity-purified antibodies. White bars show binding of antibodies that were pre-incubated with CHIPS_1–121_.

To investigate the specificity of antibodies purified on the selected peptides, a synthetic peptide from the group 1 consensus sequence (MNKSYTI) was synthesized. As the N-terminus of CHIPS was shown to contain linear epitopes for human antibodies, a N-terminal peptide (aa 1–38) of CHIPS was used for comparison. The synthesized peptides were designed to contain a C-terminal cysteine residue that allowed immobilization by thiol coupling chemistry. They were subsequently coupled to activated Sepharose to create affinity columns, which were used to capture peptide-specific antibodies from the human polyclonal IgG pool (IV-IgG). Binding of the affinity-purified anti-peptide antibodies to the CHIPS_1–121 _molecule was studied by ELISA and Surface Plasmon Resonance (SPR) (Figure 4). The affinity-purified anti-group 1 peptide antibodies, the anti-CHIPS ΔN/C antibodies as well as affinity-purified anti-1–38 peptide antibodies were found to bind to the native CHIPS_1–121 _protein in ELISA and SPR. Preincubation with CHIPS_1–121 _abolished this binding. Without affinity purification, the same concentration of IV-IgG showed no detectable binding.

### Potential antibody epitopes were identified on the CHIPS surface

To identify potential antibody epitopes, the consensus sequences 'MNKT/Sxxx' and 'GKLPxxx', were manually mapped onto the surface of CHIPS_31–121 _by structural analysis. The fold of N-terminally truncated CHIPS consists of an N-terminal amphipathic α-helix and a four-stranded anti-parallel β-sheet as determined by multi-dimensional NMR [25]. The structure of CHIPS with C-terminal truncation is not yet determined, consequently the available NMR structure was used for mapping of the epitopes. More specifically, the suggested epitopes are composed of adjacent amino acids found in the 3D structure (or amino acids with similar chemical properties to those in the consensus sequences).

In total, seven different mapping possibilities of the two consensus sequences were identified. These are denoted Surface 1.1–1.6 and 2.1 (Table 2). Residue-residue distances between Cα, Cβ or the closest side chain atoms of the epitope were measured to verify that the amino acids building up the proposed epitopes were adjacent (See Additional files: Table 1).

**Table 2:** Suggested anti-CHIPS ΔN/C IgG epitopes.

***Consensus group 1***	**M**	**N**	**K**	**T/S**	**x**	**x**	**x**
Surface 1.1		N55	K100	T53	S107	Y108	
Surface 1.2		N55	K100	S107	Y108		
Surface 1.3		Q58	K100	S107	Y108		
Surface 1.4		N55	K54	T53	Y108		
Surface 1.5		N68	K69	G70	Y71	Y72	
Surface 1.6		N111	K95	Y94	Y97	Y71	

***Consensus group 2***	**G**	**K**	**L**	**P**	**x**	**x**	**x**

Surface 2.1		K69	L90	P35	K92	E67	

**Figure 5:**
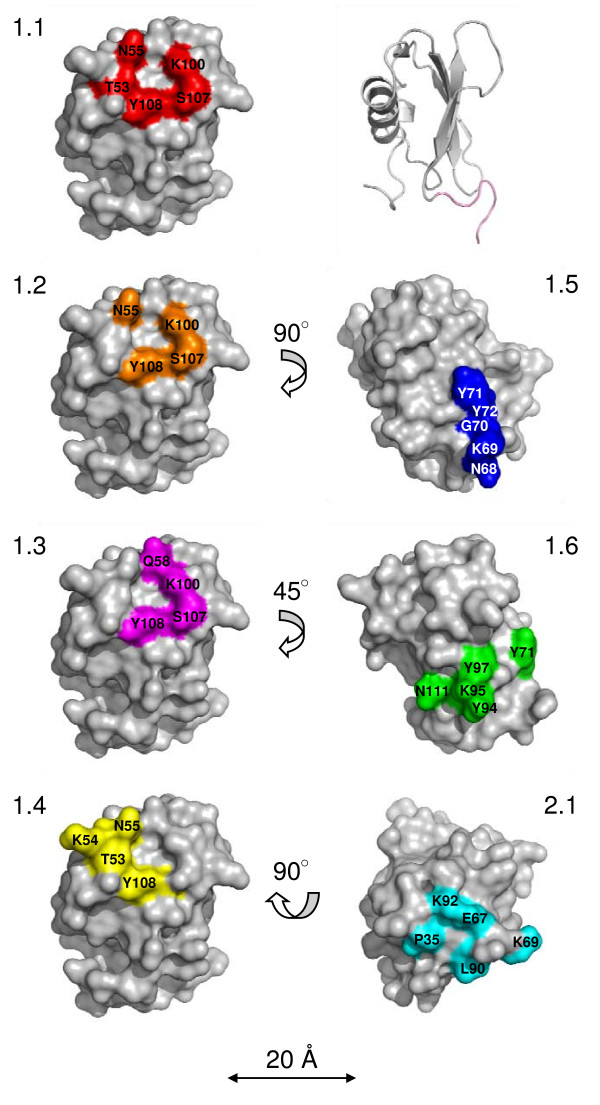
**Conformational antibody epitopes in the CHIPS_31–121 _structure**. Surface representation of N-terminally truncated CHIPS (PDB code: 1XEE) with potential conformational antibody epitopes shown in different colors; surface 1.1 (red), surface 1.2 (orange), surface 1.3 (magenta), surface 1.4 (yellow), surface 1.5 (blue), surface 1.6 (green) and surface 2.1 (cyan). Figures were generated by the PyMOL molecular graphics system [37].

Surfaces 1.1–1.4 are based on the Group 1 consensus sequence and are largely overlapping, differing only in one or two amino acids (Figure 5). Surface 1.5 and 1.6 are also based on the Group 1 consensus sequence, but they are situated in a different part of the protein and are composed of different amino acids. Only one mapping possibility was found for the Group 2 consensus sequence. This potential epitope (Surface 2.1) was situated on the opposite side of the CHIPS molecule as compared to Surfaces 1.1–1.4.

### Verification of proposed surfaces by mutational analysis

In an attempt to verify the proposed surfaces and their relation to antibody epitopes, a mutational strategy was applied. To produce mutants with key amino acids altered, point mutations (one per sequence) were introduced into the C-terminally truncated CHIPS sequence. The specific amino acid substitutions were designed to alter the suggested epitopes by substituting key amino acids to residues with different chemical properties or size. In addition, the specific amino acid substitutions were also chosen in a way not to destabilize the protein. Hence, similar amino acids have been substituted to different amino acid types depending on their location in the protein. However, not all amino acids were suitable for mutagenesis, as they were predicted to be important for preserving the structure of CHIPS. These amino acids, R46 [25] as well as Y72, L76 and Y108 (with side-chains protruding into the interior cavity of the protein), were therefore left unchanged.

The CHIPS mutants were characterized in a CHIPS capture ELISA for decreased binding of human anti-CHIPS ΔN/C IgG (Table 3) by making full titration curves of the mutants. The cut-off value for decreased binding was set to 90% of maximum binding of the wt C-terminally truncated CHIPS binding curve. 9 out of the 17 mutants showed a decreased reactivity with human anti-CHIPS ΔN/C IgG and are marked in bold in Table 3. Each of the seven mapped surfaces, defining potential conformational epitopes in CHIPS, involved one to three residues that one by one were important for antibody binding. Residues K100, S107 and N55 were found to be involved in three suggested epitopes (K100 and S107: Surface 1.1–1.3) (N55: Surface 1.1, 1.2 and 1.4). Other amino acids found to be important for antibody binding were N68, K69, K92, K95, Y97 and N111. K69 was represented in both Surface 1.5 and 2.1, whilst the other residues were only represented in one of the proposed surfaces.

**Table 3:** Suggested anti-CHIPS ΔN/C IgG epitopes and amino acid substitutions that have been made to verify epitopes*.

***Consensus group 1***	**M**	**N**	**K**	**T/S**	**x**	**x**	**x**
Surface 1.1		**N55K**	**K100A**	T53G	**S107N**	Y108	
mutations		**86%**	**78%**	93%	**76%**	-	
							
Surface 1.2		**N55K**	**K100A**	**S107N**	Y108		
mutations		**86%**	**78%**	**76%**	-		
							
Surface 1.3		Q58K	**K100A**	**S107N**	Y108		
mutations		90%	**78%**	**76%**	-		
							
Surface 1.4		**N55K**	K54R	T53G	Y108		
mutations		**86%**	90%	93%	-		
							
Surface 1.5		**N68A**	**K69T**	G70	Y71	Y72	
mutations		**80%**	**88%**	-	96%	-	
							
Surface 1.6		**N111K**	**K95S**	Y94H	**Y97K**	Y71K	
mutations		**78%**	**47%**	97%	**49%**	96%	

***Consensus group 2***	**G**	**K**	**L**	**P**	**x**	**x**	**x**

Surface 2.1		**K69T**	L90E	P35A	**K92E**	E67K	
mutations		**88%**	92%	97%	**85%**	91%	

* The suggested epitopes are denoted Surface 1.1–1.6 and 2.1.

Binding of anti-CHIPS ΔN/C IgG to CHIPS variants was determined by ELISA and is shown as % binding to anti-CHIPS ΔN/C IgG in the table. Nine unique CHIPS variants with a maximum binding lower than 90% of wt CHIPS were found. These are marked in bold and were selected for further characterizations. In some positions no substitutions were made. This is marked by -.

### New CHIPS variants can still block activation of the C5aR

**Figure 6:**
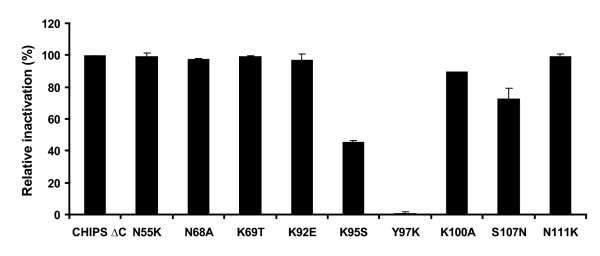
**Inhibition of C5aR activity by CHIPS variants with C-terminal truncation**. Fluo-3AM labeled U937/C5aR transfectants were preincubated with CHIPS variants and stimulated with C5a. Results are expressed as percentage inhibition of buffer treated cells.

To investigate whether the selected new CHIPS variants with reduced antibody binding were still functional in preventing C5a induced activation of the C5aR, the 9 mutants were purified and analyzed in a calcium flux assay. Serial dilutions of the variants were preincubated with Fluo-3AM labeled U937/C5aR cells and the cells were stimulated with 1 nM C5a. Flow cytometry data showed that mutants N55K, N68A, K69T, K92E and N111K retained full C5aR blocking activity at 1 μg/ml. In contrast, Y97K completely abolished and K95S, K100A and S107N partially abolished the biological activity in comparison to wt C-terminally truncated CHIPS (Figure 6). Thus, some mutations that interfere with antibody binding are not compatible with biological function.

### Structural comparisons using circular dichroism spectroscopy (CD)

**Figure 7:**
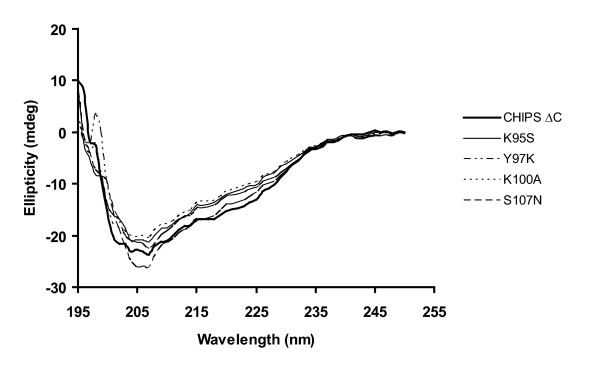
**CHIPS with C-terminal truncation and selected corresponding mutants have the same overall structure**. CD spectra of C-terminally truncated CHIPS and variants with reduced C5aR blocking activity, carrying one of the amino acid substitutions K95S, Y97K, K100A or S107N, were recorded. All CHIPS variants have a significant absorption minimum at 205 nm and are suggested to have the same overall structure as the corresponding wt protein.

The K95S, Y97K, K100A and S107N mutants showed reduced C5aR blocking activity. In order to ascertain if this reduced activity was due to mutation of C5aR contacting residues and not major conformational changes compared to the structure of the corresponding wt, CD spectra for the new CHIPS variants were investigated. Even spectra of variants with decreased biological activity showed a significant absorption minimum at 205 nm similar to the corresponding wt. These introduced mutations consequently are unlikely to have affected the global fold of the molecule (Figure 7).

## Discussion

Antibodies often react with tertiary structures formed through protein folding. In contrast to linear epitopes, these epitopes are formed when remote amino acids in the primary sequence of a protein are brought into close proximity in the folded protein, hence they are referred to as discontinuous or conformational epitopes. Antibody screening of phage-displayed peptide libraries is an efficient method to identify both linear and conformational epitopes [21,22]. Epitopes have been revealed both for monoclonal [28,29] and polyclonal antibodies [24,30,31]. In this study, we used two phage-displayed random peptide libraries to map the epitopes for polyclonal human anti-CHIPS ΔN/C IgG on CHIPS. Phage selection depends on many factors, for instance, arginines in the displayed peptide sequence interfere with secretion of the coat protein pIII and consequently clones with peptides containing arginines are strongly selected against [32]. Furthermore, the stringency and nature of the wash steps can favor certain phages [33]. To increase the specificity of selected phages for binding to CHIPS IgG, we used competition elution with a high concentration of CHIPS in the last round of selection.

Peptides found to be involved in human anti-CHIPS ΔN/C IgG binding all originated from the phage library which expressed linear peptides, and not from the library with cyclic peptides. We speculate that linear peptides might have been favorable in these selections since they may be able to adopt more conformations than the more rigid cyclic peptides. Mapping of the linear peptides obtained from phage selections with anti-CHIPS ΔN/C IgG identified seven potential epitope surfaces, out of which six represent conformational epitopes. This is in accordance with the result from the pepscan ELISA, where mainly peptides spanning the N-terminal part of CHIPS were recognized by anti-CHIPS IgG. Antibody binding to CHIPS is thus likely to be a combination of interaction with conformational and linear epitopes.

The interaction between an antibody and its epitope is dependent on several amino acid residues [34]. The nature of the amino acid substituted in a mutagenesis study can affect the ability to detect its involvement in the investigated epitope [35]. Here, we have substituted residues of the suggested epitopes by amino acids with different characteristics in an effort to detect the epitopes recognized by polyclonal anti-CHIPS ΔN/C IgG. Furthermore, single mutations are likely to affect the binding of only a fraction of all antibodies in a polyclonal preparation. Therefore, the effect of site-directed mutagenesis of a single residue may be difficult to detect by ELISA, as was found in this study. If the mutations mediating an effect on antibody binding were combined in one sequence they may have a synergistic effect to further decrease the antibody interaction with CHIPS.

Nevertheless, we were able to identify amino acid residues important for antibody interaction. By mutagenesis of residues in the different proposed surfaces, we retrieved information on which of the suggested surfaces were likely to be true epitopes. Surface 1.2 and 1.6 are the surfaces affected most by the mutagenesis. For surface 1.2, mutagenesis in three out of three positions resulted in lower interaction with the IgG. N55 in the loop between the α-helix and β1, K100 in the β3 sheet and S107 located in the short loop between β3 and β4 were all shown to be involved in antibody interaction. Surface 1.6 contains two amino acids (K95 and Y97 in the β3 sheet) that upon mutagenesis reduced the antibody binding to less than 50%, respectively. N111, located in a well defined long loop between the β2 and β3 strands, was also shown to contribute to antibody binding. Surface 1.1–1.4 are overlapping structures. Surface 1.1, 1.3 and 1.4 were considered less likely to be true epitopes since mutagenesis of amino acids T53 in surface 1.1 and 1.4, Q58 in 1.3 as well as T53 and K54 in 1.4 did not affect the binding to the IgG. Surface 1.5 was another alternative epitope originating from the group 1 consensus sequence. This was the only linear epitope suggested, but the relevance of this surface was difficult to verify, since two of the five proposed residues (G70 and Y72) were not mutated. G70 and Y72 were considered important for maintaining the structure and were not mutated for stability reasons. Surface 2.1 was the only mapping possibility found for the group 2 consensus sequence. We consider this to be a less likely epitope, since mutagenesis of its amino acids did not reduce the antibody binding to a similar extent as that observed for 1.2 or 1.6.

During the mutational analysis for verification of antibody epitopes, amino acids involved in the interaction with the C5aR were also identified. The β3 sheet is comprised of amino acids Y94-K100 and is most likely critical for binding to the C5aR since we show that substitutions K95S, Y97K and K100A as well as S107N affect the C5aR inhibiting activity of CHIPS, while leaving the CHIPS structure intact. These data confirm previous findings where the substitution K95A was implicated in C5aR blocking activity [25]. Some of the amino acid residues found to be important for the antibody interaction are therefore also most likely important for the receptor binding properties of CHIPS. This corroborates data from Wright *et al. *who recently showed that CHIPS-specific antibodies from human serum could inhibit the binding and activity of CHIPS to the C5aR [20].

The data about which amino acids are involved in C5aR binding is a finding that may be utilized in the design of an optimal CHIPS molecule that requires retained C5aR binding and blocking in addition to minimal binding of human antibodies. Our study suggests residues that must be left untouched or substituted in a more conservative manner. For certain applications, there may also be other aspects to consider, such as analysis of the presence of T-cell epitopes. Such a process can be added in future studies when designing new CHIPS variants.

## Conclusion

In conclusion, selection of peptide displaying phage libraries using a pool of CHIPS specific polyclonal human IgG resulted in the identification of peptide sequences that were mapped onto the CHIPS surface to form conformational antibody epitopes. These were verified by mutational analysis and at least two epitopes likely to be true epitopes were characterized. Amino acids important for C5aR binding within these epitopes were also identified by the mutagenesis. The mapping of human antibody epitopes on CHIPS is a step towards the development of an improved CHIPS molecule. Such a C5aR antagonist with low interaction with human IgG would potentially be an effective anti-inflammatory agent.

## Methods

### Cloning, expression and purification of recombinant proteins

Full-length CHIPS_1–121 _and CHIPS with N-terminal truncation, composed of amino acids 31–121 (CHIPS ΔN) and CHIPS with N- and C-terminal truncations, composed of amino acids 31–113 (CHIPS ΔN/C) were cloned, expressed and purified as described previously [27]. CHIPS with C-terminal truncation (CHIPS ΔC) and variants thereof were cloned in a modified pRSET B vector (Invitrogen, Carlsbad, CA). As a result of cloning, this protein was 112 amino acids long with two additional non-relevant amino acids included in the C-terminal end of the expressed protein. Cultivation was performed as described by Haas *et al *[27], but the protein was purified from inclusion bodies. Briefly, the CHIPS variants were purified from *E. coli *culture by lysis of the bacteria in 1/10 culture volume binding buffer (8 M urea, 20 mM Sodium Phosphate, 500 mM NaCl, pH 7.8). After sonication and filtration, the lysate was loaded onto a HisTrap Ni-column (GE Healthcare, Uppsala, Sweden). The column was washed with binding buffer, pH 7.8, pH 6.0 and pH 5.6, respectively. CHIPS was eluted with binding buffer, pH 5.6 with 50 mM EDTA and fractions containing protein as measured by A_280 _were pooled and refolded by dialysis against 50 mM Tris-HCl, pH 8.0. The His-tag was removed as described [27].

### Site-directed mutagenesis and expression of CHIPS variants in plate format

To be able to compare many mutants of truncated CHIPS, a tag is required to capture the proteins and determine relative concentrations. However, CHIPS activity has proven to be sensitive to the addition of His-tags in both the N- and C-terminus. These have to be cleaved off before functional assays can be performed. Instead, we used a mAb (2H7) recognition site located between residue 15 and 30 within the native CHIPS N-terminus that does not interfere with C5aR blocking activity [27]. Therefore the mutations were generated in CHIPS with C-terminal truncation, leaving the N-terminal 30 amino acids untouched for recognition by the 2H7 mAb in ELISA. Site-directed mutagenesis was performed in order to introduce point mutations into the CHIPS sequence with C-terminal truncation. This was performed using the QuikChange II mutagenesis kit (Stratagene, La Jolla, CA) according to the manufacturer's recommendations. The new CHIPS variants were sequence verified and transformed into *E. coli *BL-21 Star(DE3)pLysS (Invitrogen, Carlsbad, CA) for protein expression. Variants of CHIPS with C-terminal truncation were expressed in LB medium containing 50 μg/ml ampicillin and 34 μg/ml chloramphenicol in deep-well plates. Overnight cultures were diluted 1/50 in fresh LB containing 50 μg/ml ampicillin and cultured at 37°C for 3 hours. IPTG (Isopropyl β-D-Thiogalactoside) (final concentration 0.5 mM) (BDH, Poole, UK) was added to log-phase cultures to induce protein expression followed by cultivation for three hours. Protein was prepared from *E. coli *lysates by freeze-thawing the *E. coli *pellet in a buffer consisting of PBS-0.05% Tween-20, Complete EDTA-free protease inhibitor (Roche, Basel, Switzerland), 25 U/ml Benzonase (Sigma-Aldrich, St Louis, MO) and 1 KU/ml rLysozyme (EMD Chemicals, Darmstadt, Germany). The lysates were incubated for 30 min at room temperature with shaking and centrifuged at 13,000 rpm for 15 min to remove debris and were stored at -20°C.

### Affinity purification of anti-peptide IgG and anti-CHIPS IgG

A peptide, comprising the phage derived sequence MNKSYTI, was synthesized with an additional C-terminal spacer of three glycines and a cysteine for efficient coupling. (Bio-synthesis; Lewisville TX). A 38-mer peptide comprising the N-terminal part of CHIPS (first 37 amino acids plus an additional cysteine (Pepscan, Lelystad, The Netherlands) served as control. In order to couple the peptides to a solid matrix, peptides were first reduced using agarose linked Tris(2-Carboxyethyl) Phosphine (TCEP) (Pierce, Rockford, IL) and subsequently mixed with Sulfo-Link agarose beads (Pierce, Rockford, IL) in 50 mM Tris/HCl, pH 8,3 with 5 mM EDTA for 2 hours at room temperature. Unreacted groups were blocked with L-cysteine and beads were extensively washed with coupling buffer and PBS. Small 1 ml columns were used for affinity purification of IgG from a pooled human immunoglobulin preparation for intravenous use (IV-IgG) (Sanquin, Amsterdam, The Netherlands). IgG was eluted with 0.1 M glycine-HCl buffer pH 2.9 and neutralized with 1 M Tris pH 8.0. Pooled fractions were dialyzed overnight against PBS and the purified anti-peptide IgG was stored at 4°C.

Purified CHIPS variants (full-length or CHIPS ΔN/C) were coupled to CNBr activated sepharose 4B (Amersham Biosciences, Uppsala, Sweden) and packed on a column according to manufacturer's instructions. Affinity purification was performed on an ΔKTA Prime system (Amersham Biosciences) according to the manufacturer's protocol. A total of 1 g IV-IgG (20 mg/ml) over the column. Bound human IgG was eluted with 0.1 M Glycine pH 3.0 and the pH neutralized with 1 M Tris, pH 8.0. Eluted fractions containing protein were pooled and buffer was changed to PBS on PD-10 columns (Amersham Biosciences). The affinity-purified human anti-CHIPS IgG was stored at 4°C.

### ELISA

ELISA was used for many applications throughout the study. Nunc Maxisorb clear or white 96 well plates were coated with the specific protein in PBS. Incubations were carried out in a volume of 50 μl for 1 hour at RT of not described differently, always followed by washing trice with PBS-0.05%Tween-20. Dilution buffer for all reagents was PBS-0.05%Tween-20 with 1% BSA or milk powder added.

### Anti-CHIPS and Pepscan ELISA

Microtiter plates were coated with CHIPS (1 μg/ml) overnight at 4°C. Plates were blocked with PBS-0.05%Tween-20 4% BSA and incubated with different anti-CHIPS antibodies in dilution buffer with 1% BSA. Bound antibodies were detected with goat-α-human-IgG conjugated with peroxidase (Southern, Birmingham, USA) and TMB as substrate. For peptide experiments, synthetic 25-mer CHIPS derived peptides spanning the complete CHIPS_1–121 _sequence were synthesized as described earlier [27]. Plates were coated with peptide (10 μM) overnight at 4°C and ELISA was performed as described above with IgG purified on full-length CHIPS_1–121_.

### Binding of human anti-CHIPS ΔN/C IgG to CHIPS variants with C-terminal truncation

For quantification of expressed proteins, plates were coated overnight at 4°C with 3 μg/ml monoclonal anti-CHIPS Ab 2H7 directed against a peptide of CHIPS amino acids 24–30 [27]. Plates were blocked in PBS-0.05% Tween-20 with 3% milk powder, washed and incubated with serial dilutions of lysates from CHIPS variants in dilution buffer with 1% milk powder. Binding was detected with 3 μg/ml polyclonal rabbit anti-CHIPS N-terminal IgG (IgG produced by immunization of a rabbit with a KLH-coupled synthetic peptide corresponding to CHIPS N-terminal amino acids 1–14) and goat anti-rabbit IgG-HRP (Southern Biotech, Birmingham, AL)). Super Signal ELISA Pico Chemiluminescent Substrate (Pierce, Rockford, IL) was added and luminescence was measured.

For detection of binding of human anti-CHIPS IgG to the CHIPS variants, plates were coated, blocked and incubated with *E. coli *lysates as described above. Affinity-purified human anti-CHIPS ΔN/C IgG was added and binding was detected with goat-anti-human IgG HRP (Jackson ImmunoResearch, West Grove, PA) and substrate as described above.

### Random peptide phage libraries and phage selection

The Ph.D.-7™ and Ph.D.-C7C™ libraries from New England Biolabs (Ipswich, MA) were used to map the epitopes for human IgG on the surface of the CHIPS protein.

The Ph.D.-7™ Phage Display Peptide library consists of 7-mer random linear peptides fused with a linker sequence (GGGS) to the N-terminus of the major coat protein pIII of bacteriophage M13. The randomized segment of the Ph.D.-C7C™ library is flanked by a pair of cysteine residues, which are oxidized during phage assembly to a disulfide linkage, resulting in the displayed peptides being presented to the target as loops [36]. Protein G coated beads (100 μl) (Dynal, Norway) were washed three times with 1 ml PBS-0.05% Tween-20. The washed beads were blocked with 1 ml PBS-0.05% Tween-20, 5% BSA for 1 h at 22°C. Beads were washed four times and resuspended in 1 ml PBS-0.05% Tween-20. One half of the blocked beads was used for preclearing the phage stock. Therefore, 10 μl Ph.D.-7™ and 10 μl Ph.D.-C7C™ were incubated with blocked beads in PBS-0.05% Tween-20 and were incubated for 30 min at 22°C under continuous agitation. Affinity-purified human anti-CHIPS ΔN/C IgG (final concentration ~10 nM) was added to the precleared phages and incubated at 22°C for 30 min. The phage/IgG suspension was added to the remaining blocked beads and incubated at 22°C for 30 min. The beads were washed 10 times with PBS-0.05% Tween-20 to wash away unbound phages. The bound phages were eluted with 0.2 M Glycine, pH 2.2, 0.1% BSA for 8 min followed by neutralization of the pH with 1 M Tris-HCl, pH 8. The eluate was amplified and 10 μl of amplified phages was used as input for the next selection round. To increase the specificity of the phage selection, the bound phages in the fourth round were eluted using competition elution with the CHIPS protein. Bound phages were eluted by overnight incubation with 1.8 mg/ml CHIPS.

### Phage titration, amplification and characterization

LB medium was inoculated with a single colony of ER2738 *E. coli *and incubated at 37°C with vigorous shaking until mid-log phase (OD_600 _~0.5). Top agar (50% LB agar, 50% LB medium) was melted and cooled to approximately 45°C. Melted top agar (3 ml) was added to 200 μl ER2738 *E. coli *and poured on top of a LB/0.5 mM IPTG/80 μg/ml X-gal plate. 10 μl of 10-fold dilutions in LB medium was spotted on the prepared culture plates and incubated overnight at 37°C. The next day, plaques were counted in order to calculate phage titers. The remaining phage eluate was added to ER2738 *E. coli *culture at early log phase (OD_600 _0.4–0.5) and incubated with vigorous shaking at 37°C for 4.5 h. Cultured cells were pelleted at 4°C and phages were precipitated overnight at 4°C in 25% PEG6000 (Sigma-Aldrich, St Louis, MO) 3 M NaCl. The precipitated phages were centrifuged for 15 min at 10,000 rpm, 4°C. The pellet containing the amplified phages was resuspended in 200 μl PBS and titrated as described above. After the fourth selection round no phage amplification was performed and phages were directly characterized by DNA sequencing.

48 different plaques from the titration plates were stabbed with a pipette tip and used to infect 1 ml 1/100 diluted overnight culture of ER2738 *E. coli *and incubated for 4.5~5 h at 37°C. Cultures were centrifuged and 500 μl of the supernatant was precipitated with PEG6000, 3 M NaCl for 10 min at 22°C. The samples were centrifuged for 10 min at 13,600 rpm and the pellet was resuspended in 100 μl 4 M NaI, 10 mM EDTA, pH 8. 250 μl 95% EtOH was added and the samples were incubated for 10 min at 22°C to preferentially precipitate the single-stranded phage DNA. Samples were centrifuged for 10 min at 13,600 rpm and the pellet was washed with 70% EtOH, dried and sent for sequencing to MWG Biotech (Martinsried, Germany) using the PIII-96seq primer (New England Biolabs, Ipswich, MA).

A phage ELISA was performed to test the binding specificity of the selected phages for affinity-purified human anti-CHIPS ΔN/C IgG. Affinity-purified human anti-CHIPS ΔN/C IgG was coated overnight with at 4°C. Coated and non-coated wells were blocked with PBS-0.05% Tween-20, 5% BSA. Serial dilutions of the purified phage stocks in dilution buffer with 1% BSA were added and incubated for 1 h at 37°C. Detection was performed with mouse anti-M13-mAb (1 μg/ml) (Amersham Biosciences, Uppsala, Sweden) and rabbit anti-mouse IgG-HRP (Dako A/S, Glostrup, Denmark) and OPD substrate (O-phenylenediamine) (Sigma Aldrich, St Louis, MO).

### Analysis of peptide antibody binding to CHIPS

Binding of affinity-purified antibodies and pooled human IgG (IV-IgG) to CHIPS was studied by ELISA as described for anti-CHIPS ΔN/C antibodies and by SPR on a Biacore 1000 instrument. 20 μl 1 mg/ml CHIPS was directly coupled to an activated N-ethyl-N'(di-methylaminopropyl)carbodiimide (EDC) and N-hydroxysuccinimide (NHS) carboxymethyl dextran sensor chip (CM5). Unreacted groups were blocked by injection of 50 μl 1 M ethanolamine-HCl pH 8.5. Binding assays were performed at a constant flow rate of 5 μl/min at 25°C. Affinity-purified antibodies and IV-IgG were diluted in HBS-EP buffer (10 mM HEPES (pH 7.4) containing 150 mM NaCl, 3 mM EDTA and 0.005% surfactant P20). Antibodies were allowed to interact with CHIPS for 210 s followed by a 120 s dissociation phase. Additionally, the antibodies were preincubated with 1 mg/ml CHIPS protein to study competition. Affinity-purified anti-peptide antibodies were tested at a concentration of 10 μg/ml. Residual bound antibody was removed from the sensor chip surface by washing the chip for three minutes with 10 mM glycine-HCl (pH 1.5).

### Epitope mapping

The amino acid sequences of the selected phages were aligned using the Clustal-W alignment tool . Consensus sequences were manually mapped onto the surface of the CHIPS protein using the CHIPS_31–121 _NMR structure (PDB code: 1XEE) [25] and the PyMOL molecular graphics system [37].

### Biological activity assay

The C5aR inhibiting capacity of the purified CHIPS variants with C-terminal truncation was tested by measuring calcium release from C5a stimulated U937 cells stably transfected with the C5aR [1]. U937 cells (human promonocytic cell line) transfected with the C5aR (U937/C5aR) were a generous gift from Dr. E. Prossnitz (University of New Mexico, Albuquerque, NM). Cells were grown in 75 cm^2 ^cell culture flasks in a 5% CO_2 _incubator at 37°C and were maintained in RPMI 1640 medium with L-glutamine (Cambrex, Verviers, Belgium) and 10% FBS (Cambrex, Verviers, Belgium). Briefly, 5 × 10^6^/ml U937/C5aR cells were incubated with 2 μM Fluo-3AM (Sigma Aldrich, St Louis, MO) in RPMI 1640 medium with 0.05% BSA for 30 min at room temperature. After washing, the cells were preincubated with buffer or a 3-fold dilution series of CHIPS variants (1 to 0.003 μg/ml) at room temperature for 30 min. Every sample was measured on a FACSsort flow cytometer (BD Biosciences, San José, CA) and C5a (Sigma Aldrich, St Louis, MO) (final concentration 1 nM) was added. The cell population was gated on forward and side scatter and results are expressed as percentage inhibition of calcium release as compared to cells without addition of CHIPS.

### CD spectroscopy

The structures of the purified C-terminally truncated CHIPS variants were compared by the use of CD spectroscopy. The experiment was carried out on a Jasco J-720 spectropolarimeter (Jasco Inc., Easton, MD) in a 2 mm cuvette at a protein concentration of ~20 μM. Spectra were recorded from 195 to 250 nm at 20°C, at a scan speed 50 nm/min, a time constant of 4 s, a bandwidth of 1 nm, resolution of 1 nm and sensitivity of 50 mdeg.

### Data analysis

Data from ELISA and cellular experiments were plotted by the use of the Prism 4 software package (GraphPad Software Inc.). The cellular data were fitted in a nonlinear regression model (sigmoidal dose-response curve with variable slope).

## Abbreviations

CHIPS: Chemotaxis inhibitory protein of *Staphylococcus aureus*; C5a: complement fragment C5a; C5aR: C5a receptor; FPR: Formylated peptide receptor; Wt: Wild-type; SPR: Surface Plasmon Resonance.

## Authors' contributions

EG carried out the mutational analysis, functional assays and structural comparisons, participated in the design of the study and purification of antibodies and drafted the manuscript. PJH carried out the phage selections and pepscan experiments and participated in the design of the study and the epitope mapping and drafted the manuscript. BW carried out the epitope mapping and participated in the design of the study and helped to draft the manuscript. MH carried out the SPR measurements. CF participated in the design of the study and helped to draft the manuscript. MO participated in the design of the study and helped to draft the manuscript. JvS participated in the design of the study. KvK participated in the design of the study, purification of antibodies and draft of the manuscript. All authors read and approved the final manuscript.

## Supplementary Material

Additional file 1 **Additional table 1.** Additional table 1 shows the residue – residue distances (Å) between proposed epitopes. Click here for file
