# Supplementary material for: Identification of conformational epitopes for human IgG on Chemotaxis inhibitory protein of Staphylococcus aureus
Source: BMC Immunol. 2009 Mar 11;10:13. doi: 10.1186/1471-2172-10-13 (PMC2662796; doi:10.1186/1471-2172-10-13)
Supplement: Additional file 1 — Additional table 1. Additional table 1 shows the residue – residue distances (Å) between proposed epitopes. [file 1471-2172-10-13-S1.doc]

ADDITIONAL TABLE 1

**Additional table 1. Residue – residue distances (Å) between proposed epitopes.**

Distances between Cα, Cβ or the closest side chain atoms are shown.

| **Epitope 1.1** | **N55-K100** | **K100-T53** | **T53-S107** | **S107-Y108** |
| --- | --- | --- | --- | --- |
| Cα | 9.7 | 13.0 | 12.8 | 3.8 |
| Cβ | 8.4 | 11.1 | 11.2 | 5.4 |
| Closest | 7.1 | 8.4 | 10.2 | 3.8 |
| **Epitope 1.2** | **N55-K100** | **K100-S107** | **S107-Y108** |  |
| Cα | 9.7 | 7.9 | 3.8 |  |
| Cβ | 8.4 | 6.4 | 5.4 |  |
| Closest | 7.1 | 2.7 | 3.8 |  |
| **Epitope 1.3** | **Q58-K100** | **K100- S107** | **S107-Y108** |  |
| Cα | 5.4 | 7.9 | 3.8 |  |
| Cβ | 5.0 | 6.4 | 5.4 |  |
| Closest | 3.7 | 2.7 | 3.8 |  |
| **Epitope 1.4** | **N55-K54** | **K54-T53** | **T53-Y108** |  |
| Cα | 3.9 | 3.7 | 10.0 |  |
| Cβ | 5.5 | 5.2 | 7.6 |  |
| Closest | 3.9 | 3.7 | 4.1 |  |
| **Epitope 1.5** | **N68-K69** | **K69-G70** | **G70-Y71** | **Y71-Y72** |
| Cα | 3.8 | 3.8 | 3.8 | 3.8 |
| Cβ | 4.8 | - | - | 5.9 |
| Closest | 3.8 | 3.8 | 3.8 | 3.8 |
| **Epitope 1.6** | **N111-K95** | **K95-Y94** | **Y94-Y97** | **Y97-Y71** |
| Cα | 4.4 | 3.8 | 9.9 | 10.8 |
| Cβ | 5.1 | 4.7 | 10.3 | 10.7 |
| Closest | 4.3 | 3.8 | 4.8 | 7.7 |
| **Epitope 2.1** | **K69-L90** | **L90-P35** | **P35-K92** | **K92-E67** |
| Cα | 7.4 | 7.0 | 5.3 | 6.2 |
| Cβ | 6.7 | 7.4 | 6.2 | 5.8 |
| Closest | 5.5 | 5.8 | 4.9 | 2.9 |
